# Hesperetin Attenuates T-2 Toxin-Induced Chondrocyte Injury by Inhibiting the p38 MAPK Signaling Pathway

**DOI:** 10.3390/nu16183107

**Published:** 2024-09-14

**Authors:** Chunqing Lu, Wenjing Yang, Fang Chu, Sheng Wang, Yi Ji, Zhipeng Liu, Hao Yu, Shaoxiao Qin, Dianjun Sun, Zhe Jiao, Hongna Sun

**Affiliations:** 1 Institute for Endemic Fluorosis Control, Center for Endemic Disease Control, Chinese Center for Disease Control and Prevention, National Health Commission Key Laboratory of Etiology and Epidemiology, Harbin Medical University, Harbin 150081, China; lcq2020@hrbmu.edu.cn (C.L.); 202001064@hrbmu.edu.cn (W.Y.); 2020020144@hrbmu.edu.cn (F.C.); 18347807511@163.com (S.W.); 2022020105@hrbmu.edu.cn (Z.L.); m2011527395@163.com (H.Y.); 2023020211@hrbmu.edu.cn (S.Q.); hrbmusdj@163.com (D.S.); 2 Heilongjiang Provincial Key Laboratory of Trace Elements and Human Health & Key Laboratory of Etiology and Epidemiology, Education Bureau of Heilongjiang Province, Harbin Medical University, Harbin 150081, China; jiyi0728@outlook.com; 3 Institute of Keshan Disease, Chinese Center for Disease Control and Prevention, Harbin Medical University, Harbin 150081, China; 4 Institute for Kashin Beck Disease Control and Prevention, Chinese Center for Disease Control and Prevention, Harbin Medical University, Harbin 150081, China

**Keywords:** hesperetin, T-2 toxin, Kashin–Beck disease, cartilage

## Abstract

Background: Hesperetin, a flavonoid derived from citrus fruits, exhibits potent antioxidant and anti-inflammatory activities and has been implicated in cartilage protection. However, its effectiveness against T-2 toxin-induced knee cartilage damage remains unclear. Methods: In this study, high-throughput sequencing analysis was employed to identify the key signaling pathways involved in T-2 toxin-induced articular cartilage damage in rats. Animal models were divided into the following groups: control, low-dose T-2 toxin, high-dose T-2 toxin, T-2 toxin + hesperetin, hesperetin, and vehicle. Pathological staining and immunohistochemistry were used to assess pathological changes, as well as the expression levels of the cartilage matrix-related proteins MMP13 and collagen II, along with the activation of the p38 MAPK signaling pathway. Additionally, primary rat chondrocytes were cultured to establish an in vitro model for investigating the underlying mechanism. Results: High-throughput sequencing analysis revealed the involvement of the MAPK signaling pathway in T-2 toxin-induced articular cartilage damage in rats. Hesperetin intervention in T-2 toxin-exposed rats attenuated pathological cartilage damage. Immunohistochemistry results demonstrated a significant reduction in collagen II protein expression in the high-dose T-2 toxin group (*p* < 0.01), accompanied by a significant increase in MMP13 protein expression (*p* < 0.01). In both the articular cartilage and the epiphyseal plate, the T-2 toxin + hesperetin group exhibited significantly higher collagen II protein expression than the high-dose T-2 toxin group (*p* < 0.05), along with significantly lower MMP13 protein expression (*p* < 0.05). Hesperetin inhibited the over-activation of the p38/MEF2C signaling axis induced by T-2 toxin in primary rat chondrocytes. Compared to the T-2 toxin group, the T-2 toxin + hesperetin group showed significantly reduced phosphorylation levels of p38 and protein expression levels of MEF2C (*p* < 0.001 or *p* < 0.05). Moreover, the T-2 toxin + hesperetin group exhibited a significant decrease in MMP13 protein expression (*p* < 0.05) and a significant increase in collagen II protein expression (*p* < 0.01) compared to the T-2 toxin group. Conclusions: T-2 toxin activates the p38 MAPK signaling pathway, causing knee cartilage damage in rats. Treatment with hesperetin inhibits the p38/MEF2C signaling axis, regulates collagen II and MMP13 protein expression, and reduces cartilage injury significantly.

## 1. Introduction

T-2 toxin, a mycotoxin produced by Fusarium fungi, is commonly detected in moldy grains, corn, barley, and other agricultural commodities. This potent toxin poses significant health risks to both humans and animals, capable of inducing a spectrum of acute and chronic poisoning symptoms [1]. T-2 toxin has a specific affinity for articular cartilage, where it exerts profound effects such as inhibiting proliferation, damaging the extracellular matrix, inducing apoptosis, triggering inflammation, and disrupting metabolic processes. These actions can culminate in structural and functional aberrations within cartilage tissue, ultimately compromising normal joint mobility and homeostasis [2]. T-2 toxin is recognized as one of the primary pathogenic contributors to Kashin–Beck disease (KBD) [3]. Pathological alterations in KBD primarily involve multifocal degeneration and necrosis of the epiphyseal and articular cartilage, leading to secondary degenerative osteoarthropathy. Primary clinical presentations include pain, swelling, deformity, restricted mobility, and muscular atrophy in the limbs and joints [4]. KBD predominantly impacts Asian populations, with over 170,000 cases reported in China alone as of 2021 [5]. Current therapeutic approaches for KBD predominantly revolve around symptomatic relief and pain management, with a noticeable dearth of targeted treatment modalities. Therefore, the discovery of safer and more effective alternative treatment drugs is crucial for improving the quality of life for KBD patients.

Flavonoids, a class of natural products primarily found in plants, possess various biological properties, including antioxidant, anti-inflammatory, and anti-tumor activities. Studies have revealed that flavonoids also exhibit protective effects on bone health, promoting bone tissue regeneration and inhibiting osteoclast activity [6,7]. Hesperetin, a flavonoid primarily sourced from citrus peel, exhibits anti-inflammatory and antioxidant properties, supports bone cell metabolism, and demonstrates a protective capacity in conditions such as osteoarthritis and osteoporosis [8]. Research indicates that hesperetin can inhibit osteoclastogenesis and ameliorate bone loss induced by lipopolysaccharide [9]. By inhibiting NF-κB/Akt signaling pathways, hesperetin safeguards chondrocytes from apoptosis and inflammation [10,11]. Moreover, it has been observed to restore chondrocyte-triggered inflammatory responses by activating the AMPK signaling pathway, thereby inhibiting cartilage degradation, enhancing chondrocyte proliferation, and conferring cartilage protection [12]. With its potential therapeutic benefits, hesperetin emerges as a promising natural agent for osteoarthritis management. However, its efficacy in mitigating T-2 toxin-induced chondrocyte injury remains unexplored.

This study aims to establish an animal model of T-2 toxin exposure and employ high-throughput sequencing to explore the molecular regulatory mechanisms underlying T-2 toxin-induced cartilage injury. Additionally, animal and cell models were utilized to investigate the protective effects of hesperetin on T-2 toxin-induced cartilage injury both in vivo and in vitro. The findings of this study are of significant importance for understanding the mechanisms of cartilage damage induced by T-2 toxin and exploring hesperetin as a potential intervention.

## 2. Materials and Methods

### 2.1. Establishment of Animal Models

Sixty three-week-old SPF-grade SD male rats weighing 55 g ± 10 g were obtained from Beijing Weitong Lihua Laboratory Animal Technology Co., Ltd. (Beijing, China). [SCXK (Beijing) 2016-0011]. The rats were housed in the SPF Laboratory Animal Center of Harbin Medical University, with an average indoor temperature of 22 ± 2 °C and relative humidity maintained at 40–60%. After a week of adaptive feeding, the rats were randomly divided into 6 groups, with 10 rats in each group, and treated with T-2 toxin (MSS1023-1, Pribolab, Qingdao, China) and hesperetin (HY-N0168, MedChemExpress, Shanghai, China). T-2 toxin and hesperetin were dissolved in absolute ethanol, thickened with CMC-Na (viscosity: 800–1200 mpa.s, HY-Y0703, MedChemExpress, Shanghai, China), diluted to the appropriate treatment concentrations with normal saline, sonicated, and prepared as a uniform suspension. All interventions were administered orally at a dose of 5 mL/kg. The rats were divided into 6 groups: control group (normal saline), low-dose T-2 toxin (0.1 mg/kg bw/day), high-dose T-2 toxin (0.2 mg/kg bw/day), T-2 toxin + hesperetin group (0.2 mg/kg bw/day T-2 toxin, 50 mg/kg bw/day hesperetin), hesperetin group (50 mg/kg bw/day), and vehicle group. Throughout the 4-week duration of the experiment, all rats were given food and water ad libitum. The dosage for this study was established by referencing the literature [13,14,15] and findings from prior experiments. The reagent preparation method was also guided by the existing literature [16].

At the end of the experiment, the rats were sacrificed by intraperitoneal injection of 10% chloral hydrate and 40 μg/kg alfentanil hydrochloride, and the hind limbs of the rats were collected. Knee cartilage was scraped into a 1.5 mL centrifuge tube using a disposable surgical blade, and small steel beads and RIPA lysate buffer (P0013, Beyotime, Shanghai, China) or Trizol (Cat#9109, TaKaRa, Osaka, Japan) were added. The experimental protocol received approval from the Medical Ethics Committee of the Center for Endemic Disease Control at Harbin Medical University and followed established animal ethics guidelines.

### 2.2. High-Throughput Sequencing

Three rat knee cartilage tissues were randomly selected from the control group and the high-dose T-2 toxin group for high-throughput sequencing analysis, which was carried out by Beijing Novogene Technology Co., Ltd (Beijing, China). The process involved total RNA extraction, removal of rRNA using the Ribo-Zero^TM^ rRNA Removal Kit (Illumina, San Diego, CA, USA), and RNA fragmentation with an average fragment length of approximately 200 nt. Next, single-stranded cDNA was synthesized through reverse transcription, followed by the synthesis of double-stranded cDNA. The double-stranded cDNA was then purified and subjected to terminal repair. Primers were added, and PCR was performed for library construction based on RNA species. The quality of the library was assessed, and the samples were sequenced using the Illumina HiSeqTM 2500 sequencing platform. The raw data obtained from sequencing were then subjected to information analysis. The “DESeq2” package in R software 4.1.2 was used to identify the differentially expressed mRNAs, with thresholds of |fold change (FC)| > 1.5 and adjusted *p* < 0.05 for significance. Additionally, the “ClusterProfiler” package in R software was utilized for KEGG pathway analysis, with significant pathways identified at a *q* < 0.05.

### 2.3. Quantitative Real-Time PCR

A total of 5 mg of rat knee cartilage was added to 1 mL of Trizol (Cat#15596018, Invitrogen, Carlsbad, CA, USA) and homogenized using a pre-cooled ball mill (MM400, Retsch, Haan, Germany) at 30 Hz for 10 min. Total RNA was extracted with Trizol solution, and mRNA was reverse transcribed into cDNA using the Takara PrimeScript RT reagent kit (RR047A, Takara, Tokyo, Japan) according to the manufacturer’s instructions. The mRNA cDNA synthesis reaction conditions were as follows: incubation at 37 °C for 15 min, followed by heating at 85 °C for 5 s. For PCR amplification, the reaction mixture consisted of 5.0 µL of SYBR, 0.2 µL of ROX reference dye II, 0.4 µL each of forward and reverse primers, 3 µL of DEPC water, and 1 µL of cDNA. The primer sequences for the genes are provided in Table 1. Quantitative real-time PCR was conducted using the Applied Biosystems™ 7500 instrument (Thermo Fisher Scientific, Waltham, MA, USA) with the following cycling parameters: initial denaturation at 95 °C for 10 min, followed by 40 cycles of denaturation at 95 °C for 15 s, and annealing at 60 °C for 1 min. β-actin was used as the reference gene, and three wells were used for each sample to ensure accuracy. The expression levels of target genes were determined by calculating 2^−∆∆CT^ values.

### 2.4. Western Blot

The Western blot experiments were carried out following standard methods as described in reference [17]. Total protein was extracted from cartilage tissue or chondrocytes using the BCA Protein Assay Kit (P0012, Beyotime, Shanghai, China) to determine protein concentrations. Equal quantities of extracted protein (30 μg) were separated by 10% sodium dodecyl sulfate-polyacrylamide gel electrophoresis and transferred onto 0.22-µm PVDF membranes (MilliporeSigma, Burlington, MA, USA). PVDF membranes were blocked with 5% skim milk powder for 1 h. Antibodies against the respective target proteins were incubated overnight at 4 °C: phospho-p38 MAPK (1:1000, Cat#9211, Cell Signaling Technology, Danvers, MA, USA), MEF2C (1:1000, 10056-1-AP, Proteintech, Wuhan, China), p38 MAPK (1:1000, Cat#9212, Cell Signaling Technology, Danvers, MA, USA), collagen II (1:1000, ab34712, Abcam, Cambridge, UK), MMP13 (1:3000, ab39012, Abcam, Cambridge, UK), β-actin (1:5000, Cat#4967, Cell Signaling Technology, Danvers, MA, USA). The following day, PVDF membranes were washed three times with TBST and incubated with a secondary antibody, anti-rabbit IgG, HRP-linked antibody (1:5000, Cat #7074, Cell Signaling Technology, Danvers, MA, USA), for 1 h at room temperature. Chemiluminescence detection was performed using the ChemiDoc imaging system (ChemiDoc MP, Bio-Rad Laboratories, Hercules, CA, USA). Semiquantitative analysis of protein band intensity was conducted using the ImageJ v1.8.0 software and normalized to the intensity of the internal loading control, β-actin.

### 2.5. Histological Staining

The hind limb knee joints of the rats were fixed in 4% paraformaldehyde for 48 h, followed by immersion in a decalcifying solution that was changed weekly for 4 weeks. The decalcified knee tissues were then rinsed with running water, dehydrated, embedded in paraffin, and cut into 4 μm thick sections.

For HE staining, the sections were stained with hematoxylin (BL700B, Biosharp, Hefei, China) for 5 min, differentiated with alcohol hydrochloride for 5–10 s, immersed in tap water for 30 min until they turned blue, and then stained with 0.5% eosin (BL700B, Biosharp, Hefei, CHINA) for 1 min. For alcian blue nucleus solid red staining, the sections were first immersed in an acidified alcian blue solution (G1562, Solarbio, Beijing, China) for 3 min, followed by staining with an alcian blue staining solution (G1562, Solarbio, Beijing, China) for 30 min. They were then rinsed with running water for 5 min and counterstained with nuclear fast red staining solution (G1320, Solarbio, Beijing, China) for 5 min. After rinsing with running water for 1 min, the sections were gradually dehydrated, made transparent, and sealed with neutral resin before being observed under a microscope (BX53, Olympus, Tokyo, Japan).

### 2.6. Immunohistochemistry

The sections were dewaxed and dehydrated, incubated with 3% hydrogen peroxide for 30 min at room temperature, and then immersed in PBS for 5 min (repeated three times). Next, they were incubated with 0.1% trypsin at 37 °C for 30 min and then immersed in PBS for 5 min (repeated three times). After blocking with 5% BSA for 1 h, the sections were again immersed in PBS for 5 min (repeated three times) and then dried. The primary antibodies for collagen II (1:200, ab34712, Abcam, Cambridge, UK), MMP13 (1:200, ab39012, Abcam, Cambridge, UK), p38 MAPK (1:50, Cat#9212, Cell Signaling Technology, US), and MEF2C (1:50, 10056-1-AP, Proteintech, Wuhan, China) were applied to the sections and left to incubate overnight in a wet box. The next day, the cells were again immersed in PBS for 5 min (repeated three times), and then a polymeric HRP-labeled goat anti-rabbit IgG secondary antibody (SV-0004, Boster Bio, Wuhan, China) was added. After another round of PBS immersion, the sections were treated with SABC for 30 min at 37 °C and then immersed in PBS for 5 min (repeated three times). DAB chromogenic solution was applied for color development. Finally, the sections were counterstained with hematoxylin, dehydrated and made transparent, and sealed with resin. Under the microscope, three different fields were chosen on each histopathologic slide, and the average optical density (AOD) was calculated using Fiji (ImageJ).

### 2.7. Establishment of Cell Model

Three-day-old SD rats were used for primary cell extraction. The sacrificed pups were sterilized in a 75% alcohol solution using tracheal asphyxia. After 5 min, the cartilage tissue of the knee joint was cut into 2 mm^3^ pieces using sterile ophthalmic tools. The tissue was then washed with PBS and digested sequentially using trypsin digestion solution (C0201-100mL, Beyotime, Beijing, China) and 2 mg/mL collagenase II (C2-28-100mg, Sigma-Aldrich, St. Louis, MO, USA). Once the cartilage was digested into a uniform suspension, cell screening was performed using a 200-mesh cell strainer. The cell mass deposited at the bottom was discarded after centrifugation, and the remaining cells were resuspended and evenly seeded in the medium. The medium consisted of 10% fetal bovine serum (10099141C, GIBCO, Grand Island, NY, USA) and 90% DMEM/F12 (1:1) medium (11320033, GIBCO, Grand Island, NY, USA). The cells were passaged 1:4 every three days, and the third generation of cartilage primary cells was used for the study. The experimental groups consisted of: (1) control group; (2) T-2 toxin group; (3) T-2 toxin + BIRB796 (HY-10320, MedChemExpress, Shanghai, China) group; (4) BIRB796 group; (5) T-2 toxin + hesperetin group; (6) hesperetin group. The doses of different treatment factors were determined using a CCK8 assay.

### 2.8. CCK-8 Assay

1 × 10^4^ chondrocytes were seeded in each well of a 96-well plate, and 100 µL of complete medium was added. The cells were then cultured at 37 °C for 24 h to allow for adherence and subsequently treated with different factors. After the treatment, 10 μL of CCK-8 solution (BS350A, Biosharp, Hefei, China) and 90 μL of complete medium were added to blank wells and wells with inoculated cells. They were then incubated at 37 °C in the dark for 1 h before measuring the absorbance at 450 nm using a microplate reader. The cell survival rate was calculated using the formula: (OD value of treatment group − OD value of blank well)/(OD value of control group − OD value of blank well) × 100%. Blank wells did not contain any cells or treatment factors, treatment groups contained cells and treatment factors, and control groups contained only cells.

### 2.9. Transmission Electron Microscopy

Chondrocytes were gently digested with trypsin cell digestion solution (C0205, Beyotime, Beijing, China) without EDTA. The remaining complete medium on the cell surface was washed with PBS, and the cells were then centrifuged at 3000× *g* rpm for 3 min to obtain clean cell clusters. Initial fixation was performed by adding 1 mL of glutaraldehyde, followed by secondary fixation using 4% glutaraldehyde and 1% osmium tetroxide. The cell clusters were then dehydrated in a gradient of acetone solutions. Once dehydrated, they were embedded in Epon812 embedding agent and allowed to polymerize. Ultrathin sections were then prepared, and the ultrastructure of the chondrocytes was observed under a transmission electron microscope (HT7800, HITACHI, Tokyo, Japan) after double staining with uranyl acetate and lead citrate.

### 2.10. Statistical Analysis

SPSS 26 and GraphPad Prism 9 were used for statistical analysis of experimental data. Measurement data with normal distribution were described as mean ± SD. The *t*-test was used for comparison between two groups, and the Welch’s *t*-test was used for unequal variances. The measurement data of three or more groups with a normal distribution were analyzed using one-way analysis of variance (ANOVA), with Tukey’s multiple comparisons test or Dunnett’s T3 test used for pairwise comparisons between groups. A *p* value of less than 0.05 was considered statistically significant.

## 3. Results

### 3.1. Activation of the MAPK Signaling Pathway in Rat Cartilage Tissue by T-2 Toxin

**Figure 1:**
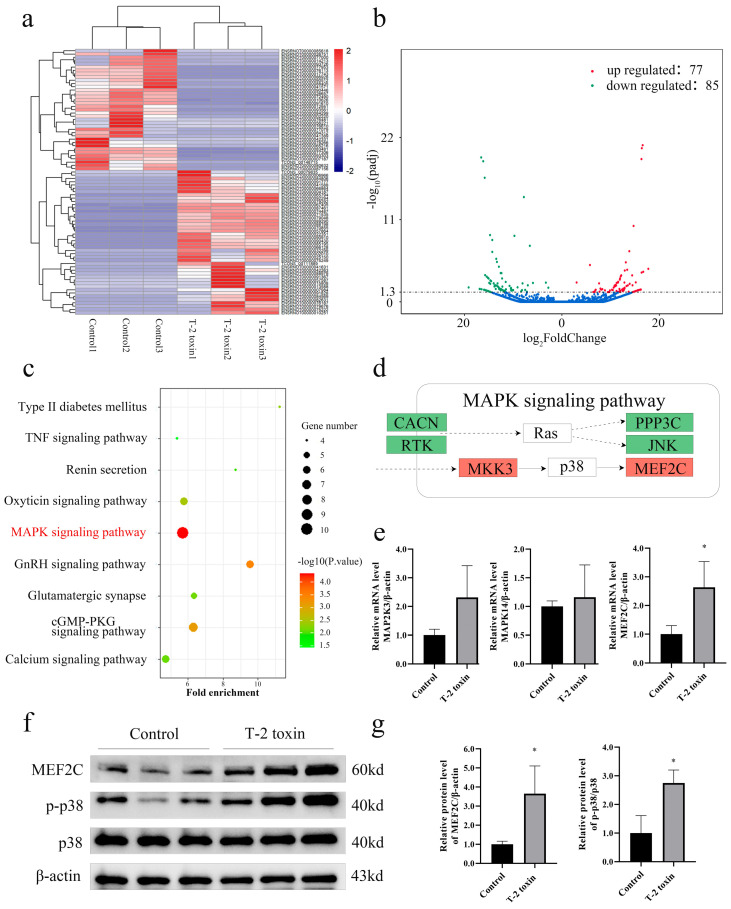
Identification of signaling pathways involved in T-2 toxin-induced cartilage damage. (**a**) Cluster heatmap depicting the expression patterns of differentially expressed mRNAs. (**b**) Volcano plot illustrating the differential expression of mRNAs. Blue dots show no expression difference from controls. (**c**) KEGG pathways analysis revealing the enriched pathways associated with differentially expressed mRNAs. (**d**) Genes enriched in the MAPK signaling pathway, highlighted in green for downregulated genes and red for upregulated genes. Dashed arrows represent indirect regulation and solid arrows represent direct regulation. (**e**) Relative mRNA expression levels of MAP2K3, MAPK14, and MEF2C in rat knee cartilage. (**f**,**g**) Western blot analysis showing the protein expression levels of p38, p-p38, and MEF2C in rat knee cartilage. Data are presented as the mean ± SD, *n* = 3, * *p* < 0.05 compared to the control group.

High-throughput sequencing revealed that, compared to the control group, the T-2 toxin-treated group exhibited significant alterations in a total of 162 mRNA molecules (Figure 1a), with 77 mRNA molecules being upregulated and 85 mRNA molecules being downregulated (Figure 1b). To assess the functional implications of these differentially expressed genes, the DAVID database was employed to conduct KEGG enrichment analysis, revealing enrichment in several pathways, including the MAPK signaling pathway, the cGMP-PKG signaling pathway, the GnRH signaling pathway, and the oxytocin signaling pathway (Figure 1c). Notably, the MAPK signaling pathway exhibited the most substantial disparity and encompassed a large number of genes. Further investigation pinpointed two differentially expressed molecules, MKK3 (gene: MAP2K3) and MEF2C, which were directly associated with the p38 MAPK signaling pathway, suggesting that this signaling axis might serve as a pivotal mechanism underlying T-2 toxin-induced chondrocyte injury.

While the mRNA expression levels of MAP2K3 and p38 (gene: MAPK14) demonstrated an increase in the T-2 toxin-treated group, the difference was not statistically significant. However, there was a significant upregulation in the mRNA expression level of MEF2C (*p* < 0.05) (Figure 1e). Subsequent Western blot analysis revealed a significant elevation in p-p38 and MEF2C expression in T-2 toxin-treated chondrocytes (*p* < 0.05) (Figure 1f,g). These findings indicate a strong association between T-2 toxin-induced chondrocyte injury and the phosphorylation level of p38 as well as the expression level of MEF2C.

### 3.2. Hesperetin Intervention Modulates Cell Count and Hypertrophic Chondrocyte Transformation in T-2 Toxin-Exposed Knee Cartilage

**Figure 2:**
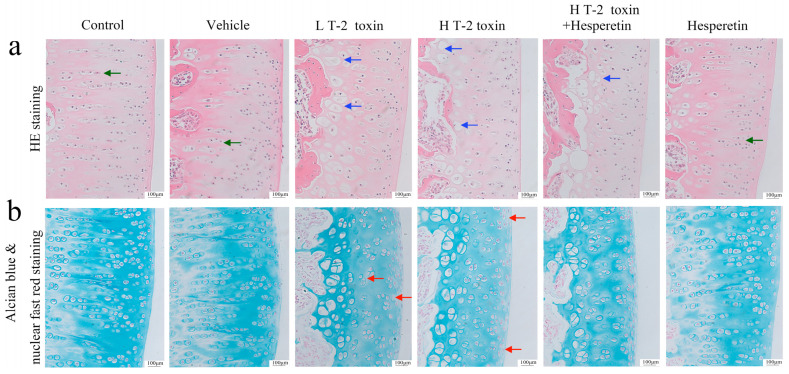
Histological analysis of articular cartilage (magnification ×200). (**a**) HE staining: green arrows indicate well-aligned columns of cells, and blue arrows indicate hypertrophic chondrocytes. (**b**) Alcian blue and nuclear fast red staining. Red arrows indicate regions of proteoglycan depletion.

HE staining was employed to examine the structural alterations of the knee joint cartilage surface. The results demonstrated that the control group exhibited a large number of chondrocytes with clear and well-arranged cell columns. Following exposure to T-2 toxin, the number of chondrocytes decreased, with some transforming into hypertrophic chondrocytes, and the cell columns became indistinct. The high-dose T-2 toxin group exhibited a more pronounced reduction in cell count. In contrast, the hesperetin intervention group showed an increase in the cell number and a decrease in the hypertrophic chondrocyte count compared to the high-dose T-2 toxin group (Figure 2a). Alcian blue staining was employed to assess changes in proteoglycan within the cartilage matrix. The outcomes revealed that the control group displayed a substantial area of blue staining on the articular cartilage, while the low-dose T-2 toxin group and high-dose T-2 toxin group exhibited loss or lightening of blue staining, indicating the depletion or reduction of proteoglycans. The hesperetin intervention group showed no significant changes compared to the high-dose T-2 toxin group (Figure 2b).

### 3.3. Hesperetin Attenuates T-2 Toxin-Induced Reduction in Collagen II and MMP13 Expression in Rat Knee Cartilage

**Figure 3:**
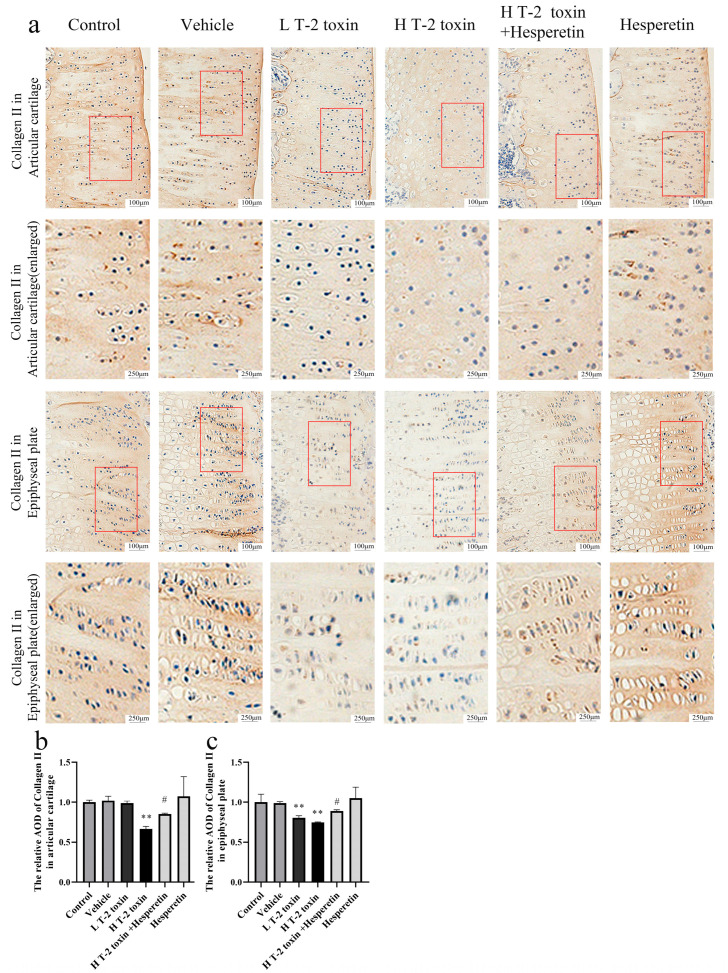
Immunohistochemical analysis of collagen II protein expression levels. (**a**) Representative immunohistochemical images illustrating the localization and staining intensity of collagen II proteins (magnification ×200 or ×500). The red frames represent selected areas that have been enlarged. (**b**) Quantification of the relative AOD of collagen II in the articular cartilage. (**c**) Quantification of the relative AOD of collagen II in the epiphyseal plate. Data are presented as the mean ± SD, *n* = 3, ** *p* < 0.01 compared to the vehicle group; ^#^ *p* < 0.05 compared to the H T-2 toxin group.

**Figure 4:**
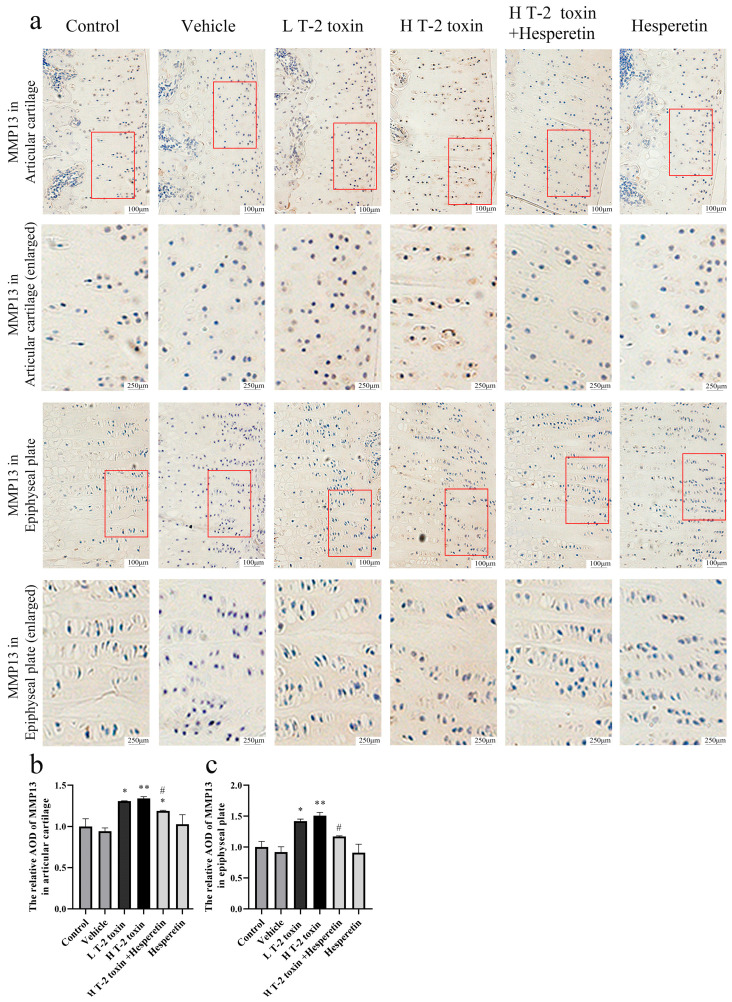
Immunohistochemical analysis of MMP13 protein expression levels. (**a**) Representative immunohistochemical images illustrating the localization and staining intensity of MMP13 proteins (magnification ×200 or ×500). The red frames represent selected areas that have been enlarged. (**b**) Quantification of the relative AOD of MMP13 in the articular cartilage. (**c**) Quantification of the relative AOD of MMP13 in the epiphyseal plate. Data are presented as the mean ± SD, *n* = 3, * *p* < 0.05, ** *p* < 0.01 compared to the vehicle group; ^#^ *p* < 0.05 compared to the H T-2 toxin group.

Collagen II is a key structural protein in cartilage, reflecting its integrity and health, while MMP13 is an enzyme involved in cartilage degradation, indicating tissue remodeling and matrix breakdown. Immunohistochemistry analysis demonstrated a significant reduction in collagen II protein expression in the articular cartilage and epiphyseal plate of rats treated with high-dose T-2 toxin compared to the vehicle group (*p* < 0.01). However, hesperetin treatment significantly increased the expression of collagen II in both the articular cartilage and epiphyseal plate compared to the high-dose T-2 toxin group (*p* < 0.05) (Figure 3). Additionally, high-dose T-2 toxin exposure led to a significant increase in MMP13 protein expression in both the articular cartilage and epiphyseal plate of rats (*p* < 0.01). Conversely, intervention with hesperetin significantly decreased MMP13 expression in the cartilage compared to the high-dose T-2 toxin exposure group (*p* < 0.05) (Figure 4). The results provide evidence that T-2 toxin induces cartilage matrix damage in rats, while hesperetin exhibits potential in mitigating T-2 toxin-induced cartilage damage.

### 3.4. Modulation of p38 and MEF2C Protein Expression in Cartilage by T-2 Toxin and the Ameliorative Role of Hesperetin

**Figure 5:**
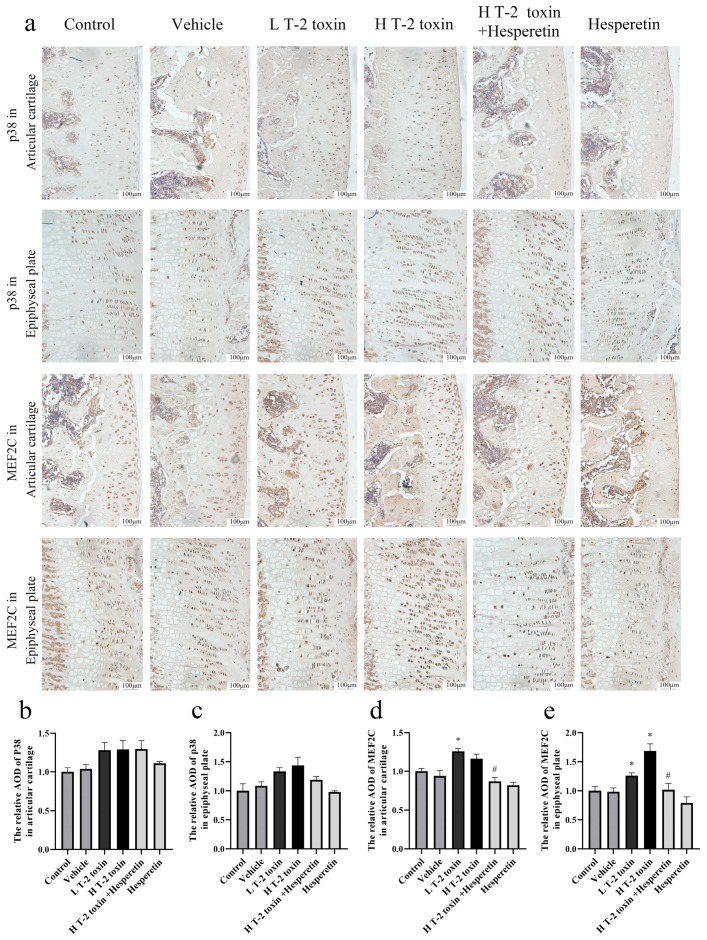
Immunohistochemical analysis of p38 and MEF2C protein expression levels. (**a**) Representative immunohistochemical images illustrating the localization and staining intensity of p38 and MEF2C proteins (magnification ×200). (**b**) Quantification of the relative AOD of p38 in the articular cartilage. (**c**) Quantification of the relative AOD of p38 in the epiphyseal plate. (**d**) Quantification of the relative AOD of MEF2C in the articular cartilage. (**e**) Quantification of the relative AOD of MEF2C in the epiphyseal plate. Data are presented as the mean ± SD, *n* = 3, * *p* < 0.05 compared to the vehicle group; ^#^ *p* < 0.05 compared to the H T-2 toxin group.

Immunohistochemistry was performed to assess the protein expression of p38 and MEF2C. The results revealed no significant difference in p38 protein expression among the groups. However, in the articular cartilage of rats, the expression of MEF2C protein was significantly higher in the low-dose T-2 toxin group compared to the vehicle group (*p* < 0.05). Similarly, in the epiphyseal plate, the expression of MEF2C protein was significantly higher in both the low-dose T-2 toxin group and the high-dose T-2 toxin group compared to the vehicle group (*p* < 0.05). Treatment with hesperetin significantly reduced MEF2C protein expression in both the articular cartilage and epiphyseal plate of rats (*p* < 0.05) (Figure 5).

### 3.5. Impact of T-2 Toxin on Viability of Primary Rat Chondrocytes and Determination of Intervention Dose

**Figure 6:**
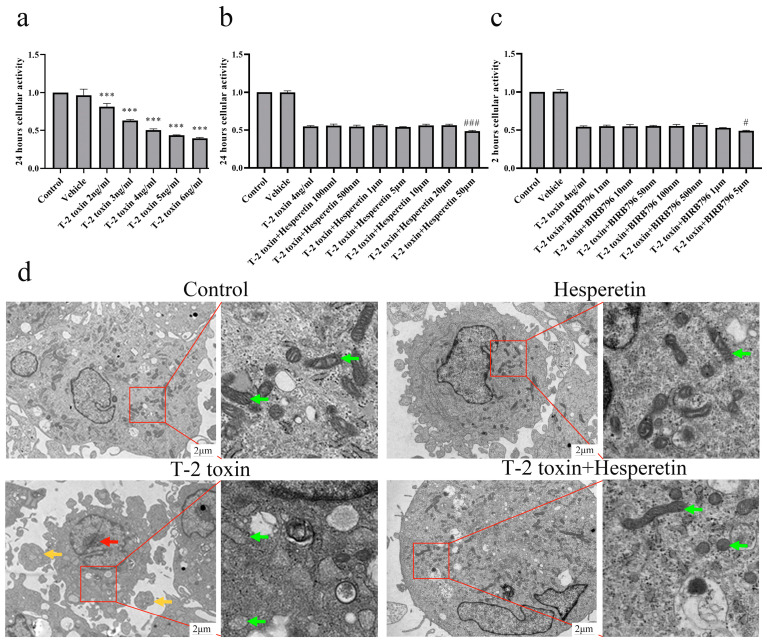
Evaluation of intervention dose and cellular ultrastructure through CCK-8 experiments and transmission electron microscopy. (**a**) Cell viability assessment after exposure to different concentrations of T-2 toxin. (**b**) Cell viability assessment after co-treatment with different concentrations of hesperetin and T-2 toxin. (**c**) Cell viability assessment after co-treatment with different concentrations of BIRB796 and T-2 toxin. (**d**) Morphology and ultrastructure of chondrocytes observed under transmission electron microscopy (magnification ×2000). Red arrow: karyopyknosis; green arrow: mitochondria; yellow arrow: apoptotic bodies. Data are presented as the mean ± SD. *** *p* < 0.001 compared to the vehicle group; ^#^ *p* < 0.05, ^###^ *p* < 0.001 compared to the 4 ng/mL T-2 toxin group.

The viability of primary rat chondrocytes, as determined by the CCK-8 assay, exhibited a significant decrease (*p* < 0.001) following treatment with T-2 toxin at concentrations ranging from 2 to 6 ng/mL for a duration of 24 h. A dose-dependent relationship was observed, with cell viability decreasing as the dose of T-2 toxin increased. When treated with 4 ng/mL T-2 toxin, the viability of primary rat chondrocytes was 46.45% (36.48–56.42%) of the control level. This value was close to 50%, which was subsequently selected as the appropriate dose for exposure in the subsequent experiments (Figure 6a). Co-treatment of primary rat chondrocytes with different concentrations of hesperetin and 4 ng/mL T-2 toxin for 24 h revealed that 50 μM hesperetin further inhibited cell viability (*p* < 0.001). Therefore, a lower concentration of 20 μM hesperetin was selected as the intervention dose (*p* > 0.05) (Figure 6b). The aim was to balance protection against T-2 toxin-induced cytotoxicity and avoid potential adverse effects of higher hesperetin concentrations (Figure 6b). Furthermore, as a p38 inhibitor, BIRB796 was found to inhibit the phosphorylation of P38. Co-treatment of primary rat chondrocytes with BIRB796 and T-2 toxin resulted in a significant inhibition of cell viability at 5 μM (*p* < 0.05). Consequently, pretreatment with 1 μM BIRB796 for 2 h was selected as the subsequent intervention dose (*p* > 0.05) (Figure 6c).

### 3.6. Cellular Characteristics and Hesperetin’s Protective Effects on T-2 Toxin-Induced Chondrocyte Damage

TEM analysis revealed distinct cellular characteristics. In the control and hesperetin groups, primary rat chondrocytes exhibited normal nuclear chromatin, intact nuclear membranes, elongated mitochondria, and dense mitochondrial cristae. Following T-2 toxin treatment, chondrocytes displayed chromatin aggregation, mitochondrial swelling, and the presence of apoptotic bodies, indicating early signs of apoptosis. However, hesperetin-treated cells showed improved nuclear chromatin distribution and reduced mitochondrial swelling, suggesting partial alleviation of T-2 toxin-induced chondrocyte damage (Figure 6d).

### 3.7. Hesperetin Modulates the p38/MEF2C Signaling Axis to Protect against T-2 Toxin-Induced Cartilage Damage

The Western blot results revealed that the T-2 toxin group exhibited a significant increase in the phosphorylation of p38 protein and upregulation of its downstream target, MEF2C protein, compared to the control group (*p* < 0.01 or *p* < 0.05). Treatment with BIRB796 resulted in a reduction in p38 phosphorylation and decreased expression of MEF2C protein (*p* < 0.05). Additionally, T-2 toxin exposure led to a decrease in collagen II content in chondrocytes and an elevation in the expression of MMP13, a protein associated with cartilage matrix degradation (*p* < 0.05), which aligned with the immunohistochemical findings in rat joint tissues (Figure 3 and Figure 4). However, intervention with BIRB796 restored the protein expression level of collagen II (*p* < 0.05). These findings suggest that targeting the p38 pathway may be a potential therapeutic approach to mitigate T-2 toxin-induced cartilage damage and degradation.

**Figure 7:**
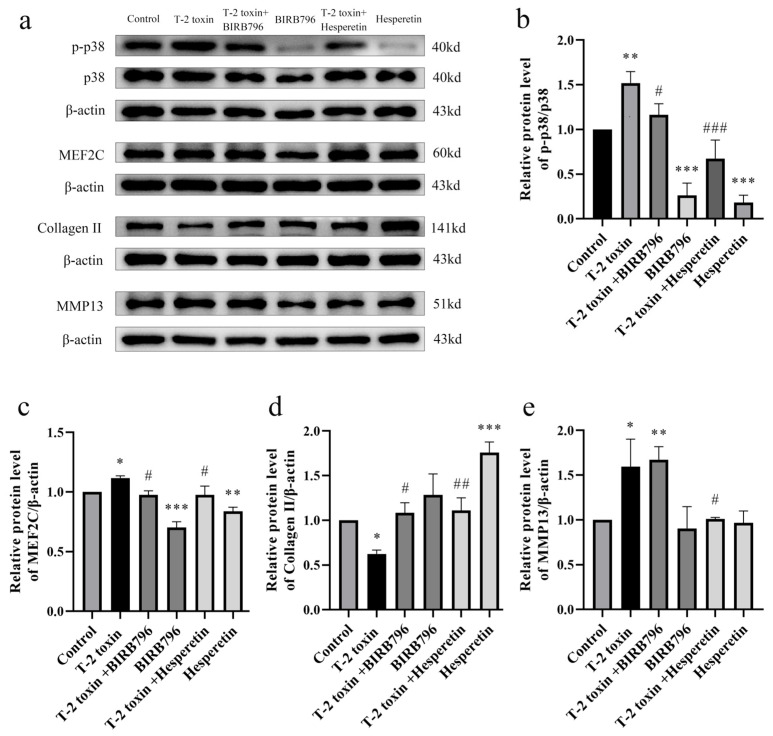
Effects of different interventions on proteins in cell models. (**a**) Representative Western blot images demonstrating protein expression. (**b**–**e**) Relative expression of proteins (p-p38, p-38, MEF2C, collagen II, and MMP13). Data are presented as the mean ± SD, *n* = 3, * *p* < 0.05, ** *p* < 0.01, *** *p* < 0.001 compared to the control group; ^#^ *p* < 0.05, ^##^ *p* < 0.01, ^###^ *p* < 0.001 compared to the T-2 toxin group.

In comparison to the T-2 toxin exposure group, the T-2 toxin + hesperetin group exhibited a significant reduction in p38 phosphorylation and MEF2C protein expression (*p* < 0.001 or *p* < 0.05). Furthermore, the protein expression level of collagen II increased, while the protein expression level of MMP13 decreased (*p* < 0.01 or *p* < 0.05). These findings indicate that hesperetin may have a protective effect against T-2 toxin-induced damage to the chondrocyte matrix by inhibiting the p38/MEF2C signaling axis (Figure 7).

## 4. Discussion

T-2 toxins are monosporin mycotoxins produced by various species of Fusarium present in moldy cereal foods and are considered to be the most toxic fungal secondary metabolites [18]. Numerous studies have demonstrated the harmful effects of T-2 toxin on chondrocytes and cartilage. Animal experiments have confirmed that feeding rats with T-2 toxin can lead to extensive necrosis of articular chondrocytes and articular cartilage defects [19]. Additionally, T-2 toxin can induce chondrocyte apoptosis and excessive degradation of the extracellular matrix [20]. Moreover, research has indicated that T-2 toxin in grains is one of the primary pathogenic factors associated with Kashin–Beck disease [3].

In this study, rats were orally administered with a dose of 0.2 mg/kg body weight per day of T-2 toxin for a duration of four weeks. The selection of this dose was based on previous studies that utilized the KBD rat model [13]. Analysis of the sequencing results demonstrated that the differentially expressed genes in the knee cartilage of rats exposed to T-2 toxin were predominantly enriched in the MAPK signaling pathway. The MAPK cascade plays a crucial role in regulating a variety of cellular processes, such as proliferation, differentiation, apoptosis, and stress responses [21]. Previous studies have indicated that the differentially expressed miRNAs observed in rats with T-2 toxin-induced cartilage injury in rats are primarily associated with apoptosis, MAPK and TGF-β signaling pathways [22]. Moreover, in cellular studies involving T-2 toxin-exposed C28/I2 chondrocytes, Yang et al. identified the MAPK signaling pathway as a key pathway involved in T-2 toxin-induced chondrocyte injury [23]. The MAPK cascade consists of four distinct signaling pathways: ERK, p38, JNK, and ERK5. Among these pathways, the p38 MAPK signaling pathway has been implicated in various cellular processes, including the induction of cell senescence, chondrocyte differentiation, synthesis of matrix metalloproteinases (MMPs), and production of pro-inflammatory factors. Inhibition of the p38 MAPK signaling pathway has shown promise in alleviating chondrocyte inflammation [24]. Guided by insights from KEGG analysis, three pivotal regulators—MKK3 (gene: MAP2K3), p38 (gene: MAPK14), and MEF2C—were selected for mRNA-level validation. Notably, among these candidates, only the mRNA expression of MEF2C, a downstream target gene of the p38 signaling pathway, exhibited a marked upregulation. Considering that the main function of p38 is mediated through its phosphorylation, we conducted Western blot analysis to assess the protein expression levels of p38, p-p38, and its downstream target MEF2C. The results demonstrated that T-2 toxin exposure increased the phosphorylation level of p38 and the protein expression of MEF2C, supporting the activation of the p38/MEF2C axis as a crucial mechanism underlying T-2 toxin-induced cartilage damage. Given the potential for multiple interactions among upstream molecules and the lack of statistically significant variances in MKK3 mRNA levels, we have excluded the upstream factor from further investigation in our study.

Hesperetin, a flavonoid compound commonly found in citrus fruits, exhibits potent antioxidant and anti-inflammatory activities. It has been demonstrated to possess protective effects in various diseases, including calcific aortic valve disease, osteoarthritis, and acute kidney injury, primarily through its antioxidant properties [12,25,26]. Being derived from natural sources, hesperetin holds promise as a sustainable therapeutic resource. Its remarkable antioxidant, anti-inflammatory, and anti-tumor activities contribute to its significant medicinal value. Research conducted by Sun Hyo Jo revealed that hesperetin inhibits microglia-mediated neuroinflammation by suppressing inflammatory cytokines and the MAPK signaling pathway, thus offering potential neuroprotection against neurodegenerative diseases [27]. Furthermore, the combined use of hesperetin and naringenin has been shown to inhibit the p38 signaling pathway, exerting a notable inhibitory effect on the growth of pancreatic cancer cells [28]. The protective effects of hesperetin on cartilage have also garnered considerable attention. Studies have demonstrated that hesperetin can inhibit bone resorption and modulate osteoblast differentiation, highlighting its potential in the field of bone health [29]. Hesperetin has been found to exhibit protective effects against osteoarthritis both in vitro and in vivo [11]. Ouyang et al. found that targeted delivery of hesperetin to cartilage via nanoparticles conferred protection to chondrocytes, thereby alleviating osteoarthritis [10]. While several studies have confirmed the beneficial effects of hesperetin on articular cartilage, the precise underlying mechanisms remain to be elucidated.

Based on the aforementioned findings, we conducted in vivo and in vitro studies to investigate the mechanism of cartilage injury induced by T-2 toxin and the protective effects of hesperetin. Previous studies have shown that Wistar rat femoral chondrocytes treated with a daily dose of 0.1 mg/kg bw/day T-2 toxin for 6 months exhibited degeneration and necrosis [14]. Additionally, Yong Li et al. reported a 14.29% positive detection rate of metaphyseal lesions in the knee joints of rats treated with a daily dose of 0.1 mg/kg bw/day T-2 toxin for 4 weeks [15]. In SD rats treated with a daily dose of 0.2 mg/kg bw/day T-2 toxin for 4 weeks, chondrocyte loss and evident necrosis of deep cartilage chondrocytes were observed [30]. Consequently, we selected two T-2 toxin doses of 0.1 mg/kg bw/day and 0.2 mg/kg bw/day for this study. The dosage of hesperetin was determined based on previous animal models, specifically the dosage used in a rat model for the protection against cisplatin-induced nephrotoxicity [31] and in an anti-inflammatory and antioxidant protection model for neuropathic pain in rats [32]. Our HE staining results also support the effectiveness of a 50 mg/kg bw/day dose of hesperetin in mitigating cartilage damage caused by a 0.2 mg/kg bw/day T-2 toxin exposure. Therefore, a dosage of 50 mg/kg bw/day of hesperetin was chosen.

Pathological examination revealed that exposure to T-2 toxin induced chondrocyte hypertrophy, alterations in cell column structure, and loss of proteoglycans in rats. These pathological changes bear similarities to the observed alterations in Kashin–Beck disease, characterized by the presence of a “red shadow” following chondrocyte disappearance and patchy loss of chondrocytes [33]. Research suggests that T-2 toxin could impact cartilage proteoglycans through cytotoxicity and metabolic disruptions, disturbing their synthesis and homeostasis, ultimately influencing cartilage structure and function [34]. Nevertheless, the intricate synthesis and maintenance of proteoglycans involve a diverse array of enzymes and signaling pathway regulations [35,36,37]. In this investigation, hesperetin intervention failed to notably reverse the decline in proteoglycan levels, possibly due to factors like dosage, treatment duration, or underlying mechanisms, warranting further exploration in future studies.

Collagen II is a complex, high molecular weight protein abundant in various amino acids. It is primarily synthesized by chondrocytes and serves as the main organic component of cartilage and joints [38]. MMP13 plays a crucial role in physiological processes such as cartilage remodeling and bone development [39]. Increased expression of MMP13 in chondrocytes can lead to the degradation of collagen II and structural damage to cartilage tissue [40]. In this study, exposure to T-2 toxin resulted in elevated expression of MMP13 and reduced expression of collagen II in the articular cartilage and epiphyseal plate of rats. These findings align with the immunohistochemistry results reported by Yi-Nan Liu [41]. Treatment with hesperetin significantly suppressed MMP13 expression and enhanced collagen II expression, confirming the ability of hesperetin to alleviate T-2 toxin-induced degradation of the cartilage matrix.

Immunohistochemistry analysis was performed to investigate the impact of T-2 toxin on the MAPK signaling pathway. Notably, high-dose exposure to T-2 toxin resulted in a significant increase in the protein expression levels of p38 and MEF2C in the epiphyseal plate. However, treatment with hesperetin effectively suppressed the expression of p38 protein and its downstream target, MEF2C. Interestingly, the observed changes in protein expression were more pronounced in the epiphyseal plate compared to the articular cartilage. This observation is consistent with the proposed pathogenesis of KBD, which primarily affects the epiphyseal plate in the epiphyseal region. One of the causes of KBD is the development of articular cartilage lesions due to exposure to T-2 toxin during the growth and development stage of children [42]. In contrast, adults who enter KBD-affected areas and are exposed to T-2 toxin typically do not exhibit severe clinical symptoms. This discrepancy can be attributed to the continuous division, proliferation, and ossification of the epiphyseal plate in children and adolescents, which allows for bone elongation and expansion. Around the age of 16 to 20, the epiphyseal plate throughout the body ceases to proliferate and ossify. This finding contributes to explaining the pathogenesis of KBD, particularly the reasons for differences in clinical manifestations after exposure between children and adults.

The selection of primary chondrocytes as the research model is based on their higher biological authenticity and specificity compared to cell lines. However, the limitations include restricted proliferation potential and genetic variation risks. To further investigate whether hesperetin exerts its protective effect by inhibiting the over-activation of the p38/MEF2C axis, we conducted experiments using primary chondrocytes. BIRB796, a p38 MAPK inhibitor capable of inhibiting all p38 isoforms [43], was chosen for pretreatment to inhibit the phosphorylation of p38. A two-hour pretreatment period prior to T-2 toxin exposure and intervention was employed. Through our investigations, we determined that a concentration of 1 μM BIRB796 effectively inhibited p38 phosphorylation without compromising cell viability.

Transmission electron microscopy analysis revealed that chondrocytes exposed to T-2 toxin exhibited mitochondrial swelling, chromatin condensation, and formation of apoptotic bodies, indicating early signs of apoptosis. Previous studies have suggested that T-2 toxin induces chondrocyte apoptosis through the endoplasmic reticulum stress pathway [20]. Additionally, Liu et al. highlighted that T-2 toxin can induce mitochondrial dysfunction, oxidative damage, and apoptosis in chondrocytes [44], which aligns with our observations. Following intervention with hesperetin, we observed a reduction in the proportion of mitochondrial swelling, decreased chromatin condensation, and the absence of apoptotic bodies. These findings suggest that hesperetin may exert a protective role in chondrocytes by inhibiting oxidative stress and apoptosis.

Hesperetin displays biphasic dose-response effects according to the hormesis principle by activating antioxidant and anti-inflammatory pathways, as well as stress response/adaptive genes for cytoprotection to prevent or restore cartilage damage and promote regeneration during toxin exposure [11]. Hormesis is a process in which small, nontoxic stresses induce adaptive responses that protect the biological system against subsequent large and potentially lethal stresses of the same, similar, or different nature [45]. This is in accordance with emerging evidence showing that low doses of hormetic nutrients such as curcumin and resveratrol upregulate antioxidant pathways, enhancing cellular stress responses to counteract free radicals and toxicity both in vitro and in vivo [46,47]. On the other hand, high doses of natural compounds can be toxic to cells and animal models, leading to the inhibition of antioxidant and anti-inflammatory pathways and the onset and progression of disorders associated with oxidative stress. In this study, a low dose of hesperetin (20 μM) was protective against T-2 toxin-induced cytotoxicity, while a high dose of hesperetin (50 μM) inhibited cell viability, resulting in toxicity in vitro. Dosage is a crucial factor in determining whether protective or harmful effects are induced and should be carefully assessed. Therefore, identifying the optimal dosage to elicit beneficial effects is of utmost importance in achieving protection and/or avoiding toxicity. The field of hormetic/adaptive responses activated by flavonoids in enhancing the endogenous redox defense systems is emerging as a promising preventive and therapeutic strategy for disorders associated with toxicity and inflammatory damage, including cartilage injury.

Exposure to T-2 toxin resulted in increased phosphorylation of p38 and elevated protein expression levels of MEF2C. MEF2C is a transcription factor that plays a crucial role in regulating muscle and cardiovascular development and is involved in controlling bone development by activating the genetic program for chondrocyte hypertrophy [48]. Increased MEF2C protein expression subsequently upregulates the expression levels of proteins associated with hypertrophic chondrocytes, such as MMP13 and Runx2 [49]. T-2 toxin exposure led to an increase in MEF2C protein expression, followed by elevated levels of MMP13 protein and a decrease in collagen II protein expression. These findings align with the research conducted by Yang Li, which demonstrated the damaging effects of T-2 toxin on rat chondrocytes [50]. Similar observations were also reported in the human chondrocyte line C28/I2 [41]. Treatment with the p38 inhibitor BIRB796 effectively mitigated the T-2 toxin-induced increase in p38 phosphorylation and MEF2C protein expression, while promoting the protein expression of collagen II. However, BIRB796 did not significantly affect the protein expression of MMP13. This could be attributed to the simultaneous inhibition of all p38 isoforms by BIRB796. Studies have indicated that p38γ, one of the isoforms, can inhibit MMP13 production in chondrocytes [51], which may explain the less pronounced effect of BIRB796 on MMP13. Hesperetin inhibited p38 phosphorylation and MEF2C expression, similar to its effects in other studies on pancreatic cancer and neuritis. It also inhibited MMP13 expression and promoted collagen II protein expression, indicating a protective role in articular cartilage by targeting the p38/MEF2C pathway.

In conclusion, our study has verified that T-2 toxin induces knee joint cartilage damage in rats through the activation of the p38 MAPK signaling pathway, with significant mitigation observed through hesperetin intervention. However, based on our high-throughput sequencing findings and existing research, T-2 toxin may also induce cartilage damage through alternative mechanisms, while reports suggest that hesperetin may engage other pathways to protect cartilage. These insights can guide future research directions. Furthermore, the potential clinical significance, efficacy, and safety of hesperetin as an intervention agent should be carefully evaluated.

## 5. Conclusions

T-2 toxin activates the p38 MAPK signaling pathway, causing knee cartilage damage in rats. Treatment with hesperetin inhibits the p38/MEF2C signaling axis, regulates collagen II and MMP13 protein expression, and reduces cartilage injury significantly.

## Figures and Tables

**Table 1:** Sequence of the primers that were used for the qPCR.

Gene	Primer Sequence
MAP2K3-F	5′-GCACTGTCGACTGCTTCTATAC-3′
MAP2K3-R	5′-GCACCTTCCGGTAGAACTTATC-3′
MAPK14-F	5′-GACATAATCCACAGGGACCTAAA-3′
MAPK14-R	5′-TAGCCGGTCATTTCGTCATC-3′
MEF2C-F	5′-AGCAGCAGCACCTACATAAC-3′
MEF2C-R	5′-GTAGAAGGCAGGGAGAGATTTG-3′
β-actin-F	5′-ACAGGATGCAGAAGGAGATTAC-3′
β-actin-R	5′-ACAGTGAGGCCAGGATAGA-3′

## Data Availability

The original contributions presented in the study are included in the article; further inquiries can be directed to the corresponding authors.
